# Impact of Natural-Based Viscosity Modifiers of Inhalation Drugs on the Dynamic Surface Properties of the Pulmonary Surfactant

**DOI:** 10.3390/ma16051975

**Published:** 2023-02-28

**Authors:** Katarzyna Dobrowolska, Małgorzata Miros, Tomasz R. Sosnowski

**Affiliations:** Faculty of Chemical and Process Engineering, Warsaw University of Technology, Waryńskiego 1, 00-645 Warsaw, Poland

**Keywords:** interfacial rheology, viscosity modifiers, biopolymers, pulmonary surfactant, air-liquid interface

## Abstract

The effectiveness of inhalation therapy depends on aerosol size distribution, which determines the penetration and regional deposition of drug in the lungs. As the size of droplets inhaled from medical nebulizers varies depending on the physicochemical properties of the nebulized liquid, it can be adjusted by adding some compounds as viscosity modifiers (VMs) of a liquid drug. Natural polysaccharides have been recently proposed for this purpose and while they are biocompatible and generally recognized as safe (GRAS), their direct influence of the pulmonary structures is unknown. This work studied the direct influence of three natural VMs (sodium hyaluronate, xanthan gum, and agar) on the surface activity of the pulmonary surfactant (PS) measured in vitro using the oscillating drop method. The results allowed for comparing the variations of the dynamic surface tension during breathing-like oscillations of the gas/liquid interface with the PS, and the viscoelastic response of this system, as reflected by the hysteresis of the surface tension. The analysis was done using quantitative parameters, i.e., stability index (SI), normalized hysteresis area (*HAn*), and loss angle (*φ*), depending on the oscillation frequency (*f*). It was also found that, typically, SI is in the range of 0.15–0.3 and increases nonlinearly with *f*, while *φ* slightly decreases. The effect of NaCl ions on the interfacial properties of PS was noted, which was usually positive for the size of hysteresis with an *HAn* value up to 2.5 mN/m. All VMs in general were shown to have only a minor effect on the dynamic interfacial properties of PS, suggesting the potential safety of the tested compounds as functional additives in medical nebulization. The results also demonstrated relationships between the parameters typically used in the analysis of PS dynamics (i.e., *HAn* and SI) and dilatational rheological properties of the interface, allowing for easier interpretation of such data.

## 1. Introduction

Nebulization is the technique of continuous generation of inhalable aerosol using drugs in the liquid phase (solutions, suspensions, and emulsions). The efficiency of such an inhalation therapy depends primarily on the particle (droplet) size distribution, which directly corresponds to the local drug deposition in the lungs [1,2,3,4]. Aerosol properties can be adjusted by nebulizer construction and the physicochemical properties of the drug formulation. For a given nebulizer, the liquid viscosity, surface tension, and, sometimes, osmolarity influence the droplet size, and thus the preferential sites of drug penetration and deposition [5,6,7,8]. Viscosity adjustment can be made by adding viscosity modifiers (VMs), which should be biocompatible and safe. We recently proposed using natural polysaccharides for this purpose [9,10]. These compounds are characterized by a high viscosity and have non-toxic, moisturizing (sodium hyaluronate), or mucoadhesive (xanthan gum and agar) properties, which are the basis for considering their potential use as additives in various medicine formulations [11,12,13]. Despite their classification as GRAS (generally recognized as safe), there is a lack of knowledge on their influence on the surface of the lungs. Following deposition in the pulmonary region of the lungs, inhaled materials meet the pulmonary surfactant (PS), which is a multicomponent system containing lipids (up to 90%, including a large proportion of phospholipids), specific proteins (up to 10%), and small amounts of carbohydrates [14]. PS forms a natural monolayer on the alveolar gas–liquid interface, forming a barrier that protects human lungs from inhalable matter [15]. These can be air contaminants [16,17,18], but also therapeutic aerosols, which need to overcome this barrier to produce the expected health effects [19,20]. Inhalable drug aerosols produced, e.g., in nebulizers, contain sufficiently small droplets to reach the alveoli [3], where they may interact with the PS. The above-mentioned PS composition determines its role in the lungs, causing periodic variations in surface tension during breathing. From an engineering perspective, the lung surface presents an elastic (or rather: viscoelastic) structure that dynamically responds to continuous variations in air pressure caused by breathing. The energy passed to the lung membrane during inspiration is not fully returned in expiration due to dissipation and partial conversion to the superficial flows, known as Marangoni effects. They are caused by locally- and time-dependent variations in the surface tension, which are macroscopically reflected by the surface tension hysteresis [21,22,23]. The significance of the Marangoni effects in mass transport, including gas exchange and the removal of deposited particulate contaminants (known as hydrodynamic self-clearance of the lungs), was widely discussed already in 1990s [21,24,25], and its role in particle toxicology was summarized in a recent review [26].

This research is focused on the investigation of the influence of selected VMs (sodium hyaluronate, xanthan gum, or agar) on the surface properties of the pulmonary surfactant during a simulated breathing cycle in experimental conditions in vitro. Dynamic surface tension data obtained by the pulsating pendant drop method are used to quantitatively assess the effects of inhaled aerosols on the lung surface and respiratory physiology. The data experimental are analyzed by quantitative criteria, also using the formalism of surface rheology. Such an analysis makes it possible to study the potential interactions of inhaled VM with pulmonary surfactants, which is an important step for evaluating their potential therapeutic applications as drug additives and may facilitate the development of new drugs for inhalation.

## 2. Materials and Methods

A multicomponent model of the pulmonary surfactant (MPS) was based on calfactant (Infasurf^®^, ONY Biotech, New York, NY, USA)—a sterile PS suspension extracted from calf lungs. It contains a mixture of lipids (phospholipids and neutral lipids) and hydrophobic surfactant-associated proteins (SP-B and SP-C). This composition is much closer to the actual physiological PS than simpler lipid models, which are often used as an experimental model of PS [27,28].

Three polysaccharides, which are categorized as GRAS compounds [29], were selected for the purpose of this study: sodium hyaluronate (SH), xanthan gum (XG), and agar (AG). A 1:1 mixture of SH with a mass greater than 1 MDa and SH with a mass less than 5 kDa and XG were prepared as follows: SH and XG were diluted with ultrapure water (MilliQ water; Merck, Rahway, NJ, USA) at room temperature and mixed overnight with a magnetic stirrer. Agar was dissolved in boiling MilliQ water and mixed overnight. Finally, the polysaccharides aqueous solutions were prepared at two concentrations: 2 and 3 mg/mL.

Infasurf^®^ was diluted with MilliQ water to a phospholipid concentration of 5 mg/mL and then mixed with VM solutions to obtain a final concentration of phospholipid at 2.5 mg/mL, which was the same as in the reference MPS sample. The final concentration of each VM equal to 0.018 mL/mL was estimated based on the calculations of the deposited mass of inhaled aerosol in the alveolar region [10].

**Figure 1:**
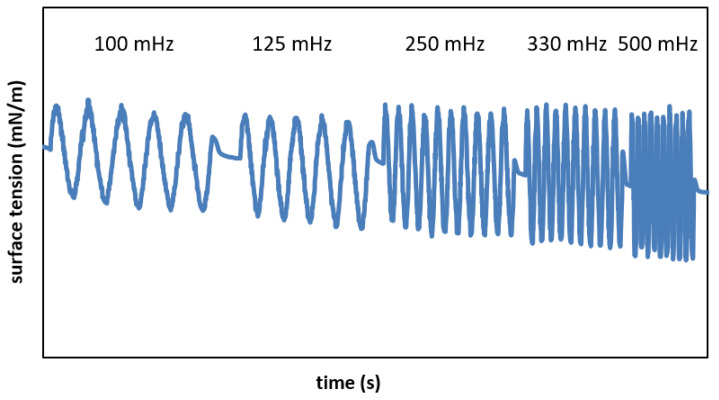
The dynamic surface tension data during surface oscillations at frequencies *f* = 100, 125, 250, 330, and 500 mHz.

Interactions between VMs and MPS were studied using the pulsating drop method in a PAT-1M drop profile tensiometer (Sinterface, Berlin, Germany) at thermostatic conditions (physiological temperature: 36.6 ± 0.3 °C). Each experiment was divided into two steps: (i) generation of a pendant drop, i.e., a new gas–liquid interface (GLI) and allowing it to equilibrate, and (ii) inducing the programmable oscillations of the drop. The first step enables monitoring changes in surface tension at a constant GLI area (A_0_ = 14 mm^2^) during system equilibration. During the second step, the measurements of dynamic surface tension were for the oscillations of the interface with 10% amplitude at a variable rate. The sinusoidal variations were done with frequencies *f* = 100, 125, 250, 330, and 500 mHz, which were selected as being comparable to the rate of human breathing at various states of physical activity [30]. Each series of oscillations was preceded by a few-seconds interval, as shown in Figure 1.

Cyclic expansion and compression of a GLI area in the presence of MPS (with or without the VMs in the liquid phase) allowed for measuring the surface tensions hysteresis and then calculating the quantitative criteria that are helpful in hysteresis characterization. As already mentioned in the Introduction, the surface tension hysteresis in the pulmonary surfactant system is the essential indicator of the physiological properties and function of PS. Two criteria are useful for characterizing the hysteresis. The stability index, *SI*, can be determined according to Equation (1) [31], where *σ_max_* and *σ_min_* denote the maximum and minimum surface tension values recorded during the oscillations, respectively. The normalized hysteresis area, *HAn*, is calculated from Equation (2), [32], where *A* denotes the instantaneous GLI area, and *A_max_*, *A_min_*—the maximum and minimum GLI area during pulsations, respectively.

(1) $$SI=\frac{\sigma_{max}-\sigma_{min}}{\frac{1}{2}\left(\sigma_{max}+\sigma_{min}\right)}$$

(2) $$HAn=\frac{{\left[\int_{A}^{}\sigma dA\right]}_{expansion}-{\left[\int_{A}^{}\sigma dA\right]}_{compresion}}{A_{max}-A_{min}}$$

The application of the Kelvin–Voigt model of surface viscoelasticity (Equation (3)) enables a deeper understanding of the processes governing the surface tension variations in the lungs during breathing using the concepts of surface rheology [30]:

(3) $$\Delta \sigma =\varepsilon_{d}\gamma +\mu_{d}\dot{\gamma }\;$$

Determining the characteristic rheological parameters of GLI, e.g., dilatational surface viscosity, *μ_d_*, and elasticity, *ε_d_*, helps in tracing changes in the surface tension variations, as well as in the surface tension hysteresis caused by dilatational deformation of the interface, *γ*, which is defined as follows:

(4) $$\gamma =\frac{dA}{A}\approx \frac{A-A_{0}}{A_{0}}$$

where *A*_0_ denotes the initial GLI area. At fixed oscillation conditions (amplitude and frequency), *μ_d_* and *ε_d_* are constant, so analyzing their changes caused by other components (such as inhaled drugs) allows for tracing the influence of these additives on the interfacial dynamics of the PS system. It is important to note that the dependence of *μ_d_* and *ε_d_* on the oscillation frequency is caused not only by the intrinsic mechanical properties of the interface with the surfactant, but also by continuous mass exchange (due to advection and diffusion) and surfactant adsorption to the GLI during expansion or desorption during compression [33].

Using Kelvin–Voigt model for oscillatory deformations, the dilatational viscosity and elasticity can be related to surface tension hysteresis via the phase (loss) angle *φ* (Equation (5)):

(5) $$\varphi =\arctan\frac{\omega \mu_{d}}{\varepsilon_{d}}$$

where the oscillation rate, *ω* [rad/s]:

(6) ω=2πf

As the loss angle shows the phase shift between the change in the interfacial area and the surface tension, Equation (5) shows that three parameters fully determine the hysteresis shape: surface elasticity, surface viscosity, and oscillation frequency/rate. When viscosity begins to dominate elasticity at a given frequency, the hysteresis width increases. A more comprehensive discussion of these relationships can be found elsewhere [30,33,34,35].

## 3. Results and Discussion

### 3.1. Characterization of Surface Tension Variations during Area Alterations

**Figure 2:**
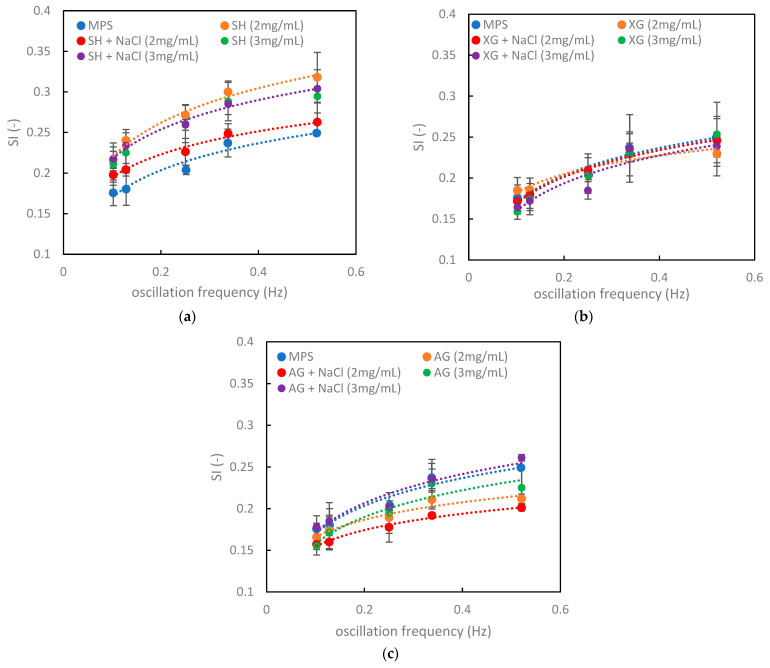
Stability index (*SI*) of the air–water or air–saline interface in the presence of MPS and VMs: (**a**) SH, (**b**) XG, and (**c**) AG.

The selected model of pulmonary surfactant (MPS) is a mixture of a milky color, which contains lipids (including phospholipids and neutral molecules, e.g., cholesterol) and proteins stabilizing this system. The stable nature of the mixture is confirmed, inter alia, by the stability index (*SI*) shown in Figure 2a–c (blue circles for pure MPS). The data are characterized by good reproducibility and a gradual smooth increase in *SI* vs. oscillation frequency. Table 1 shows both the values of the minimum surface tension, *σ_min_*, and *SI* for pure MPS. It is seen that *σ_min_* slightly decreased (from 29.6 to 27.9 mN/m) with the increasing oscillation frequency, which explains why *SI* became higher at faster oscillations (see Equation (1)).

The *SI* results for various mixtures of MPS with VMs shown in Figure 2a–c allow for discussing the influence of these biopolymers on the pulmonary surfactant. For all samples, a smooth nonlinear increase in *SI* with *f* was preserved. This was caused mainly by the expansion of the limits of the surface tension variations (decrease in σ_min_ and increase in σ_max_), which also reflect the increase in surface elasticity of GLI. The fluctuations in the instantaneous surface tensions, which were related to the presence of SH and salt ions, did not significantly affect the course of the stability index curves (Figure 2a). Only a slight increase in the *SI* value was observed (compared with pure MPS), which could be considered a positive effect regarding the possible interactions of inhaled SH with the surfactant in vivo. The deviation from MPS was practically negligible for XG (Figure 2b) and there was no role of the electrolyte (NaCl). For all types of added AG solutions, *SI* values at *f* = 0.1 Hz were grouped around the same value as for MPS, but diverged nonlinearly at higher oscillation frequencies (Figure 2c). Almost all *SI* values for AG at a given *f* were lower than for pure MPS, in contrast with what was found for SH (Figure 2a). This may suggest a slight reduction in the native dynamic properties of the surfactant by AG. However, when NaCl was present in the 3 mg/mL AG solution, *SI* was the same as for pure MPS.

**Figure 3:**
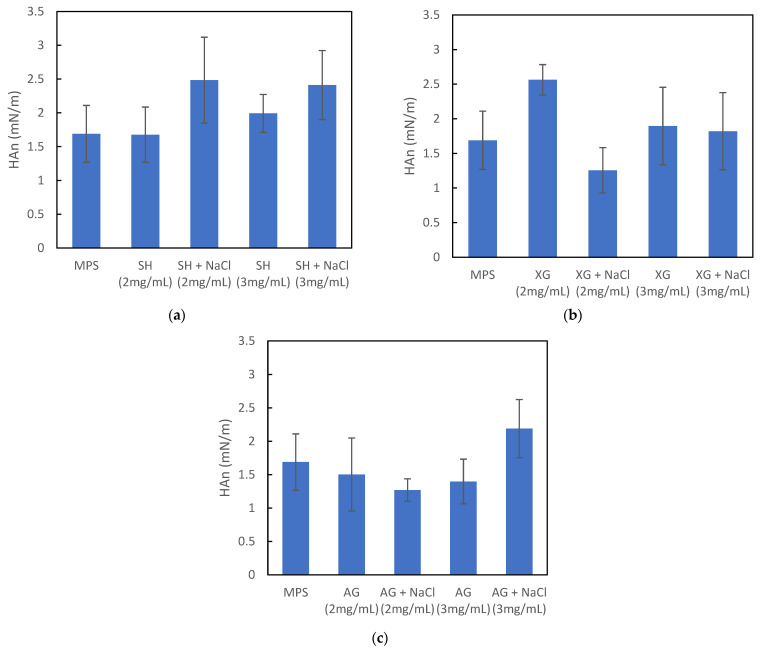
Normalized hysteresis area (*HAn*) of air–water or air–saline interface in the presence of MPS and VM: (**a**) SH, (**b**) XG, and (**c**) AG. Oscillation frequency *f* = 0.1 Hz. Error bars show the standard deviation (*n* = 6).

Figure 3 compares the normalized hysteresis area, *HAn,* calculated from *σ–A* relationships for various samples recorded at the oscillation frequency of 0.1 Hz. We observed that *HAn* was also changed in the presence of VM and salt ions. Similar tendencies were found for other frequencies (data not shown). The addition of SH (Figure 3a) increased the *HAn* value, and the presence of the electrolyte amplified this effect. The role of the SH concentration was also noticeable. Interactions between the ions and MPS components were already discussed by other researchers. For instance, it was shown that sodium cations formed a bond with the lipid carbonyls in the DPPC surface layers, while the chloride anions created so-called ionic cloud around the choline groups of this lipid [36]. It is also known that NaCl at higher concentrations not only interacts with MPS, but also affects the rheological properties of SH solutions via the alteration of spatial conformation of molecules [37,38]. These effects may explain the observed influence of NaCl on the interfacial dynamics of MPS in the presence of VMs.

The influence of XG on the *HAn* value in the MPS system is shown in Figure 3b. The pure biopolymer caused a slight increase in the hysteresis loop area, but the addition of charge carriers (ions) greatly reduced this effect. The observed phenomena could be related both to the MPS/biopolymer interactions and the spatial conformation of the XG molecules. The presence of sodium cations and chloride anions may also have an effect on the rheological properties of xanthan gum solutions. 

The addition of AG had a similar impact on the *HAn* value (Figure 3c) as XG. AG caused a narrowing of the hysteresis loop, with only one exception, when the 3 mg/mL AG solution contained NaCl. The difference between the hysteresis area of the pure MPS system and MPS in the presence of AG (3 mg/mL) with NaCl was minimal, which was similar to the negligible effect found in the *SI* relationships (Figure 2c).

The precise interpretation of the results of the VM–MPS interactions influencing the dynamic surface tension characteristics of PS is complex and difficult, because a variety of factors influence the physicochemical properties of the VMs and their behavior in the mixture with the surfactant. In addition, small amounts of substances that are the necessary additives in the inhalation drugs (e.g., isotonic substances, such as NaCl) can also modify these properties by affecting the surface charge and spatial conformation of the molecules [37,38,39]. In the case of XG, the analysis of the rheological properties of the interface is complicated by the possible role of the stiffness of the biopolymer chains, which may contribute to the additional resistance during GLI deformations [40,41,42]. As stated earlier, our studies focused on the comparative quantitative assessment of the influence of selected biopolymers on MPS in the context of the pulmonary safety of inhaled VMs. Accordingly, the validity of our findings regarding the expected consequences of the presence of the VMs in the PS system remained valid, even if the specific physicochemical mechanisms could not be precisely identified and explained based on the limited experimental data.

### 3.2. Characterization of Surface Rheological Properties in the Presence of MPS

The GLI dynamic characteristics determined during cyclic deformation around the quasi-equilibrium value of the surface tension (Figure 1) enabled finding the dilatational rheological parameters of the surface (*ε_d_* and *μ_d_* in Supplementary Materials Figure S1). These are the basic criteria for studying the dynamic properties of the deformable interface and are widely applied in the pulmonary surfactant research in vitro [30,43,44]. The typical relationship shows that surface viscosity decreased and elasticity increased at higher rates of surface deformation (i.e., higher oscillation frequency).

Both properties (*ε_d_* and *μ_d_*) combined into another surface rheological parameter—the phase angle, *φ* (Equation (5)), allow for an easy discussion of the frequency-dependent changes in the shape of the surface tension hysteresis. By analyzing the changes in *φ*, we can predict which component becomes more important and how this influences the arrangement of the compression and expansion branches on *σ-A* graphs.

The shape of the hysteresis loop and the loss angle *φ* are related to the unbalanced surface energy during surface oscillations. In physiological situations, the energy spent by the organism for lung expansion during inhalation to force the airflow into the lungs (active phase of breathing) is partly related to overcoming the surface tension forces in the alveoli. Exhalation is the passive phase of breathing, indicating that the system has a surplus of energy, which is partly manifested in the surface tension hysteresis. According to the published hypotheses, this energy can be partly converted to hydrodynamic phenomena known as interfacial convection (Marangoni effects), contributing to pulmonary mass transfer, including gas-exchange and self-clearance from particulate deposits [21,25,26]. 

**Figure 4:**
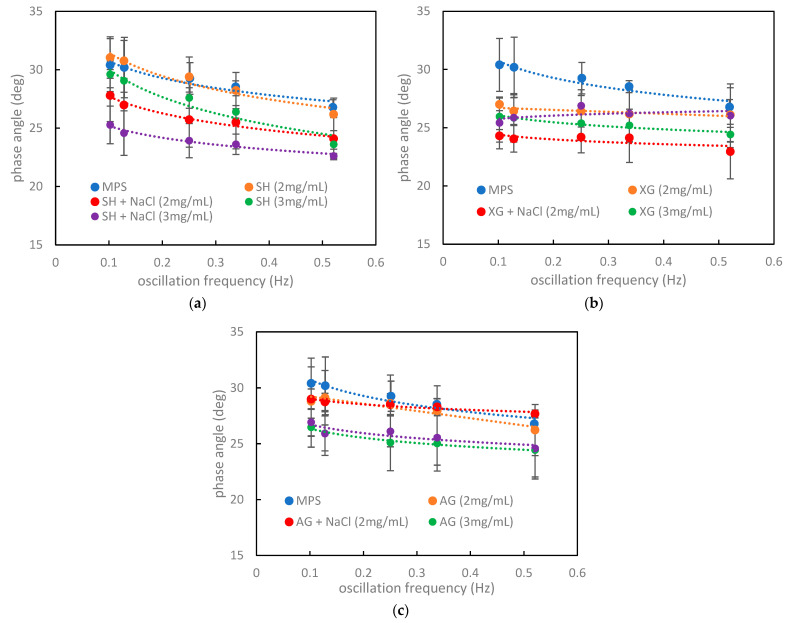
Phase angle (*φ*) of air–water or air–saline interface in the presence of MPS and VM: (**a**) SH, (**b**) XG, and (**c**) AG.

An analysis of the loss angle in the ‘pure’ MPS system (blue markers in Figure 4a–c) shows that increasing the oscillation frequency leads to a decrease in the phase angle. This trend is not surprising as the surface viscosity has a stronger decrease than the increase in elasticity when the deformation rate becomes higher.

In the presence of SH (Figure 4a), this trend is always maintained. SH also has a minor effect on the phase angle in MPS and causes only a slight decrease in *φ* at any oscillation frequency. This means that the compression and expansion branches of the hysteresis curve come closer to each other, indicating that the elastic component of the interface becomes more important than the viscous one. Therefore, we observed a less circular shape for the hysteresis compared with pure MPS.

XG (Figure 4b) had a stronger impact on the phase angle than SH. *φ* values were almost constant or slightly increased at higher deformation rates, but they were lower than for pure MPS. The oscillation frequency affected the phase angle quasi-linearly, and as with SH, there was only a slight difference between samples with and without salt ions. Unlike SH, the presence of XG caused the expansion of the hysteresis loop. This suggests more surface energy available for the above-mentioned transfer processes, hence the effect can be considered as beneficial from a physiological viewpoint.

The presence of AG in MPS caused a decrease in *φ* values at a higher biopolymer concentration. Less-concentrated solutions were characterized by slightly smaller *φ* at slow deformation rates and increased *φ* at higher oscillation frequencies (>0.33 Hz). The differences between NaCl-free solutions and saline solutions were negligible.

### 3.3. Relations between the Numerical Criteria, Rheological Parameters, and Hysteresis Shape in the Context of the Impact of Inhaled VMs on the Pulmonary Surfactant

The surface tension hysteresis can be conveniently described by numerical parameters (*HAn* and *SI*), but also by rheological criteria of the interface (*ε_d_*, *μ_d_*, and *φ*). Analyzing such quantities allows for a numerical comparison of the influence of various agents on the surface tension hysteresis. As discussed earlier, the knowledge about the arrangement of the compression and expansion branches of the hysteresis helped in assessing the correctness of physiological functions of the pulmonary surfactant under dynamic conditions of breathing. Here, we tried to answer if natural compounds potentially used as viscosity modifiers of medicines delivered by nebulization may influence these functions after deposition on the alveolar membrane, through the physicochemical interactions between these additives and PS.

The conceptual analysis of the relationships between the measured parameters, namely the phase angle and stability index, shows that the alterations of these parameters were closely related to the normalized hysteresis area *HAn* and hysteresis shape. Parameter *SI* was closely related to the amplitude of the surface tension changes and it was more dependent on the surface elasticity than on the surface viscosity at moderate surface deformation rates ($\dot{\gamma }$—Equation (3)).

**Figure 5:**
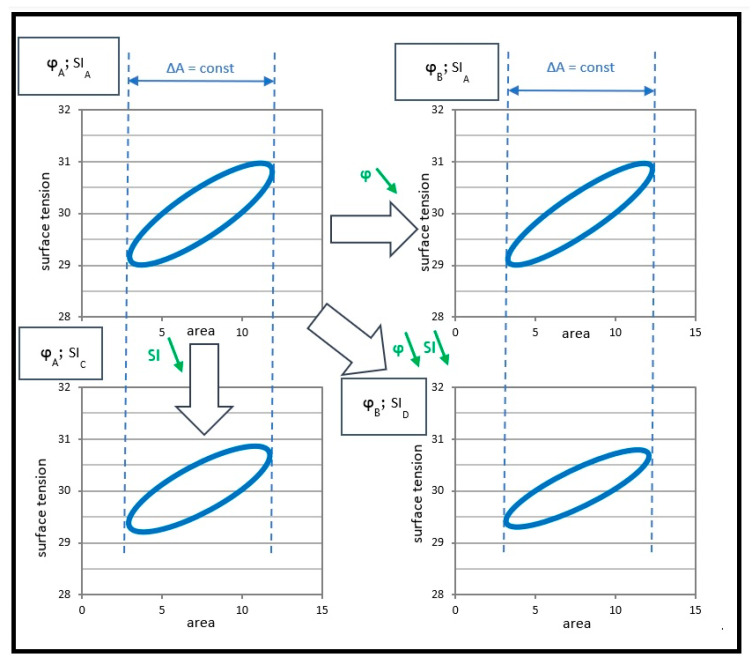
Theoretical analysis of the relationship between various numerical parameters describing *σ–A* hysteresis in an MPS system and their influence on the hysteresis shape and size.

The numerical parameters of the hysteresis calculated from *σ(A)* curves were related to each other. Figure 5 shows the general links between the discussed parameters and their relation to the shape of the hysteresis. For instance, it is seen that when *SI* decreased, *HAn* also decreased if *φ* was constant or became smaller. When *φ* was reduced, it was possible that *HAn* remained unchanged, but only for increasing *SI.*

Applying this analysis to our results of SH interacting with MPS, we can see that this compound increased the stability index by expanding the range of the minimum and maximum surface tension (*σ_max_* and *σ_min_*). On the other hand, SH impacted the rheological parameters of the surface, leading to a decreased phase angle, so there was a narrowing of the hysteresis loop. These were two opposite effects regarding the hysteresis area, reflected by the *HAn* parameter. The combined effect depended on the range of changes in each property. When the alteration in *φ* was much smaller than in *SI*, then the hysteresis area increased, and this was proven by the experimental results (Figure 3a). We can also see from our data that the presence of salt anions in the SH solution resulted in a slight decrease in *SI* and a strong increase in *φ*. As a consequence, the hysteresis area increased (also confirmed in Figure 3a).

In the case of XG, the situation was even easier to analyze. *SI* obtained at the oscillation frequency of 0.1 Hz did not change significantly in relation to the reference value (Figure 2b), with the exception of the NaCl-free solution at a biopolymer concentration of 2 mg/mL, where an increase in SI could be observed. Simultaneously, *φ* was lower than for pure MPS (Figure 4b), which was directly related to the altered *HAn*. However, as the surface deformation rate increased, the role of the rheological parameter (*φ*) was reduced. Therefore, the general tendency regarding the influence of XG on the MPS dynamics, assuming theoretical predictions, should be manifested in the narrowing of the hysteresis loop. It should be more visible at higher oscillation frequencies (>0.25 Hz). However, the presence of the electrolyte (ions) reduced the hysteresis loop even more than XG itself.

The results obtained for AG showed a similar relationship between hysteresis criteria and surface rheology, as in case of XG, with the exception of a saline solution at a biopolymer concentration of 3 mg/mL. Generally, the relationships illustrated in Figure 5 describe the results obtained for AG well. The addition of AG, irrespective of the concentration, and the contents of salt resulted in a simultaneous decrease in *φ* and *SI*, which was related to a narrowing of the surface tension hysteresis. This theoretical conclusion was confirmed by the experimental data (Figure 3c).

Taking into account all of the results presented in this study, it is possible to state that none of the studied VMs impaired the native dynamic surface-active properties of the pulmonary surfactant in a way that could indicate a possible deterioration of the surfactant functions in vivo. Minor variations in the quantitative criteria describing the surface tension hysteresis did not show any essential loss in the PS biophysical function activity, in contrast with the results obtained for many other inhalable compounds in various studies [30,45,46,47].

The advantage of this research is using the pulsating pendant drop method, which allows for experiments on a multicomponent protein-enriched pulmonary surfactant model under physiological conditions. This method requires a small volume of PS for a single measurement. An additional advantage of this method is the rapid rate of surface changes with frequencies corresponding to human respiration in different states of physical activity [30]. This is particularly important in the context of the naturally occurring phenomenon of surface tension hysteresis. On the other hand, the research method used in this study was based on certain assumptions and simplifications. The use of a multicomponent model of pulmonary surfactant, which usually yields some scatter regarding adsorption and desorption at the oscillating interface, resulted in some scatter of the results. Minor fluctuations in the results may also have resulted from the design of the measuring equipment, for example, due to droplet evaporation during measurements. Another limitation of the results is that we did not use nebulized aerosol with VM, but prepared mixtures of VM with the surfactant. This situation was different than that for aerosols deposited on the lung surface. For technical reasons, it was not possible to perform experiments with an aerosol and to assure the controlled the dose of VM added to PS. On the other hand, by mixing VM with PS, we were able to obtain the exact dose of the additive that resulted from calculating the deposited fraction of droplets generated from the actual nebulizer [10].

This work is a continuation of our earlier studies related to the influence of selected biopolymers—viscosity modifiers (VMs)—on the characteristics of inhalable aerosols delivered from nebulizers [9,10]. Those initial studies clearly showed that use of VMs could help in adjusting the properties of inhalable mist, without modification of the inhaler construction and, thus, could be used to optimize aerosol delivery to the respiratory system. The current phase of the research contains in vitro studies of interactions of VMs with the model of pulmonary surfactant, which allows for a preliminary assessment of the safety of inhaled VMs. At the same time, it provides a more complete understanding of the relation between the most often used quantitative criteria of the surface tension hysteresis and surface rheological parameters, which can be determined by dynamic surface tensiometry. The obtained results regarding the safety of VMs are promising in the context of future applications.

## 4. Conclusions

The paper presents an analysis of interactions of natural polysaccharides, proposed as viscosity modifiers of liquids used in medical nebulization, on the model of pulmonary surfactants. The analysis was done using quantitative parameters characterizing changes in the surface tension hysteresis, which are considered an important indicator of lung function within the alveolar region. It was demonstrated that none of three tested compounds (sodium hyaluronate, xanthan gum, and agar) altered the dynamic interfacial properties of the surfactant when used at concentrations relevant to their expected deposited dose in the lungs during aerosol inhalation. It suggests that each of these biopolymers may be considered as safe additives to liquid formulations for nebulization, when used to influence size distribution of the aerosol generated in nebulizers and to improve lung deposition. It was shown that possible effects of inhaled compounds on the pulmonary surface may result from physicochemical properties of a given polysaccharide, but also can depend on the presence of NaCl. Additionally, the presented analysis shows how the quantitative parameters used to characterize the shape of the surface tension hysteresis in the pulmonary surfactant are linked to each other, which facilitates the assessment of their role in the characterization of interfacial dynamics. This approach allows for finding the relationships between the viscoelastic properties of the gas–liquid interface and other quantitative criteria of the hysteresis loops, helping in a fast determination of the possible alterations of the pulmonary surfactant by inhaled agents, including drug additives.

## Figures and Tables

**Table 1:** The minimum values of surface tension, *σ_min_*, and SI for MPS at the air–water interface at different frequencies of surface oscillation.

	*f* = 100 mHz	*f* = 125 mHz	*f* = 250 mHz	*f* = 330 mHz	*f* = 500 mHz
*σ_min_* (mN/m)	29.62 ± 0.45	29.35 ± 1.14	28.67 ± 0.21	28.42 ± 1.72	27.92 ± 1.42
*SI* (-)	0.18 ± 0.02	0.18 ± 0.02	0.20 ± 0.01	0.24 ± 0.02	0.25 ± 0.00

## Data Availability

The datasets generated and analyzed during the current study are available from the corresponding author on reasonable request.

## Supplementary Materials

The following supporting information can be downloaded at: https://www.mdpi.com/article/10.3390/ma16051975/s1, Figure S1: The dilatational elasticity, ε_d_ (1) and viscosity, μ_d_ (2) air-water or air-saline interface in presence of MPS and VM: (A) SH (B) XG (C) AG.
