# Supplementary material for: Impact of Natural-Based Viscosity Modifiers of Inhalation Drugs on the Dynamic Surface Properties of the Pulmonary Surfactant
Source: Materials (Basel). 2023 Feb 28;16(5):1975. doi: 10.3390/ma16051975 (PMC10004148; doi:10.3390/ma16051975)
Supplement: Supplementary file 1 [file materials-16-01975-s001.zip › materials-2170484-supplementary.pdf]

Supplementary Materials

The determination of surface rheological parameters characterizing the dynamic state to which the air-liquid interface was introduced hold during cyclic deformation around the quasi-equilibrium surface tension. The dilatational elasticity, ε_d and viscosity, μ_d referring to the storage and loss modulus, respectively are the basic criteria for testing the dynamic properties of the deformable interface. ε_d and μ_d are commonly used in *in vitro* studies of pulmonary surfactants as the viscoelastic GLI properties enable tracking alterations of dynamic surface tension and hysteresis σ-A. A typical relationship shows that surface viscosity decreases and elasticity increases at higher surface deformation rates (i.e. higher oscillation frequency) (Figure S1).

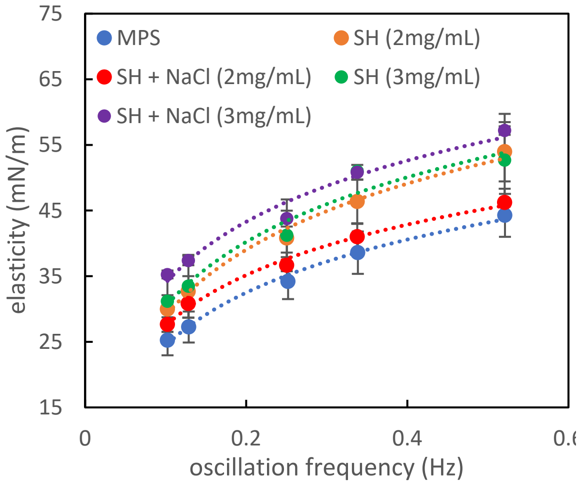

(A1)

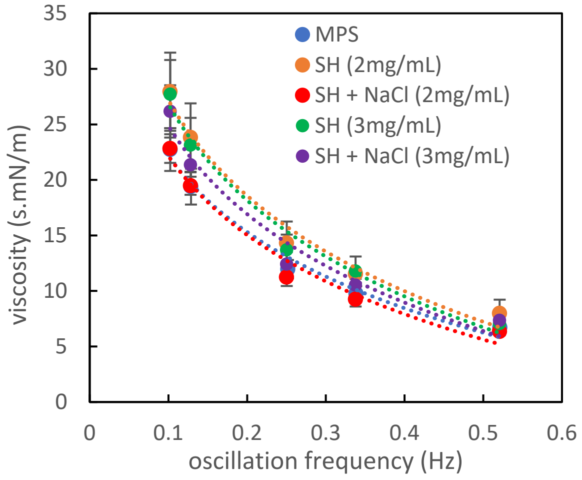

(A2)

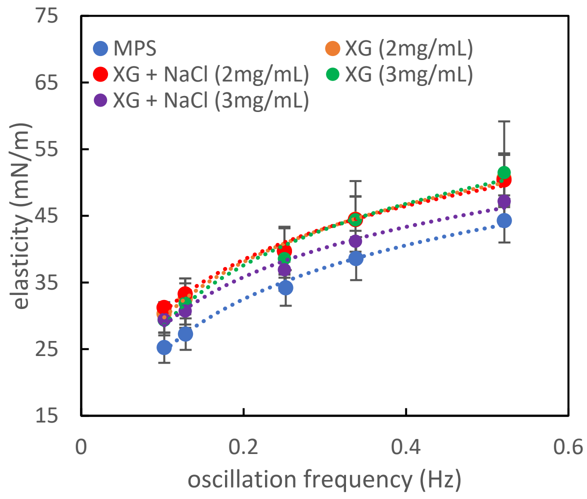

(B1)

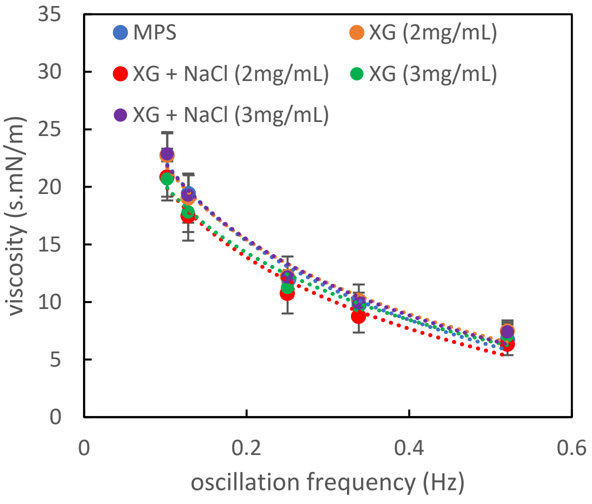

(B2)

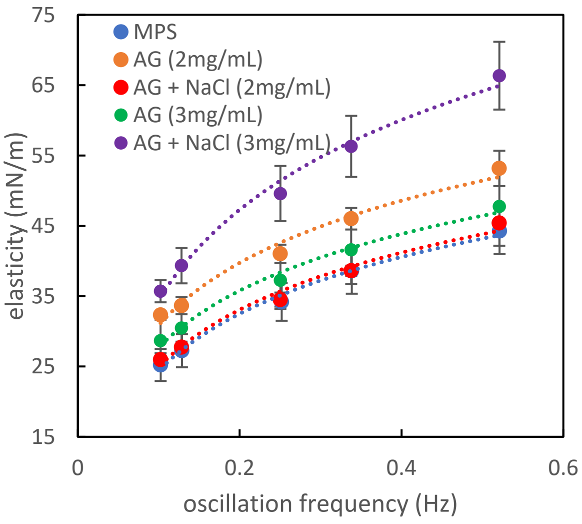

(C1)

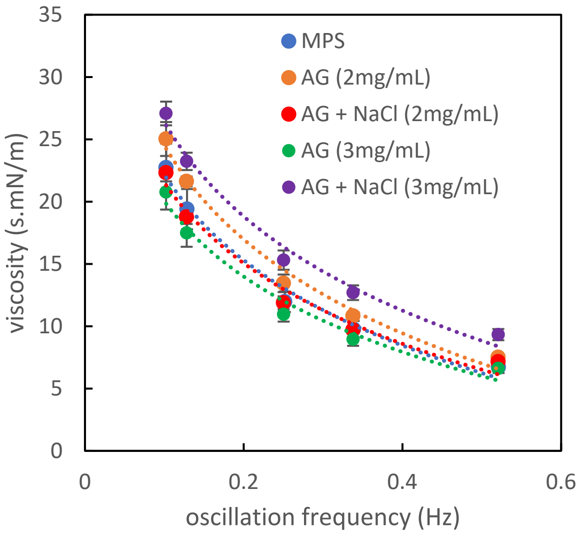

(C2)

Figure S1. The dilatational elasticity, ε_d (1) and viscosity, μ_d (2) of air-water or air-saline interface in presence of MPS and VM: (**A1, A2**) SH (**B1, B2**) XG (**C1-C2**) AG.
